# *Vibrio cholerae* Cytolysin Causes an Inflammatory Response in Human Intestinal Epithelial Cells That Is Modulated by the PrtV Protease

**DOI:** 10.1371/journal.pone.0007806

**Published:** 2009-11-12

**Authors:** Gangwei Ou, Pramod Kumar Rompikuntal, Aziz Bitar, Barbro Lindmark, Karolis Vaitkevicius, Sun Nyunt Wai, Marie-Louise Hammarström

**Affiliations:** 1 Department of Clinical Microbiology/Immunology, Umeå University, Umeå, Sweden; 2 Department of Molecular Biology, Umeå University, Umeå, Sweden; Columbia University, United States of America

## Abstract

**Background:**

*Vibrio cholerae* is the causal intestinal pathogen of the diarrheal disease cholera. It secretes the protease PrtV, which protects the bacterium from invertebrate predators but reduces the ability of *Vibrio*-secreted factor(s) to induce interleukin-8 (IL-8) production by human intestinal epithelial cells. The aim was to identify the secreted component(s) of *V. cholerae* that induces an epithelial inflammatory response and to define whether it is a substrate for PrtV.

**Methodology/Principal Findings:**

Culture supernatants of wild type *V. cholerae* O1 strain C6706, its derivatives and pure *V. cholerae* cytolysin (VCC) were analyzed for the capacity to induce changes in cytokine mRNA expression levels, IL-8 and tumor necrosis factor-α (TNF-α) secretion, permeability and cell viability when added to the apical side of polarized tight monolayer T84 cells used as an *in vitro* model for human intestinal epithelium. Culture supernatants were also analyzed for hemolytic activity and for the presence of PrtV and VCC by immunoblot analysis.

**Conclusions/Significance:**

We suggest that VCC is capable of causing an inflammatory response characterized by increased permeability and production of IL-8 and TNF-α in tight monolayers. Pure VCC at a concentration of 160 ng/ml caused an inflammatory response that reached the magnitude of that caused by *Vibrio*-secreted factors, while higher concentrations caused epithelial cell death. The inflammatory response was totally abolished by treatment with PrtV. The findings suggest that low doses of VCC initiate a local immune defense reaction while high doses lead to intestinal epithelial lesions. Furthermore, VCC is indeed a substrate for PrtV and PrtV seems to execute an environment-dependent modulation of the activity of VCC that may be the cause of *V. cholerae* reactogenicity.

## Introduction

*Vibrio cholerae*, the causal organism of the diarrheal disease cholera, is a Gram-negative motile bacterium acquired via intake of contaminated food or water. Secreted cholera toxin (CTX) is the major factor that causes diarrhea. However, most *V. cholerae* vaccine candidates lacking CTX still exhibit reactogenicity in clinical trials [1]–[4]. The molecular and cellular mechanisms behind these adverse effects are still unknown. Levine et al. [5] suggested that a previously unidentified enterotoxin could cause symptoms in the absence of CTX, but its existence has been masked by the activity of CTX in the virulent strains. Proposed candidates are the Hap protease [6], [7], the multifunctional autoprocessing RTX toxin [8], and *Vibrio cholera* cytolysin (VCC), also called hemolysin A and El Tor hemolysin [9].

Using reverse molecular genetics in the *V. cholerae* O1 strain C6706, we showed that a previously uncharacterized extracellular Zn^2+^-binding metalloprotease, PrtV, confers protection against grazing by natural bacteriovorous predators such as flagellates and ciliates [10], [11]. Furthermore, PrtV was found to be essential for bacterial killing of the nematode *Caenorhabditis elegans*, a phenomenon associated with colonization of the *C. elegans* intestine [10]. We hypothesized that the PrtV protease also actively contributes to the gut inflammation seen in volunteers in cholera vaccine trials, possibly by inducing an inflammatory response with secretion of the chemokine interleukin-8 (IL-8) by epithelial cells. However, by using polarized tight monolayers of the intestinal epithelial cell line T84 as an *in vitro* model for human intestinal epithelium, our results suggested that PrtV is not an IL-8 stimulator in man, but instead reduces the IL-8-inducing activity of an as yet unidentified secreted component(s) of *V. cholerae* [10]. In order to understand the intriguing contrasting effects of PrtV in man compared to invertebrates, we aimed at identifying secreted component(s) of *V. cholerae* that induces an epithelial inflammatory response and define whether the active component(s) is a substrate for PrtV.

## Results

### Luria-Bertani (LB) Culture Supernatants of *V. cholerae* C6706 PrtV Deletion Mutants Cause an IL-8-Dominated Inflammatory Response in Human Intestinal Epithelial Cells

**Figure 1:**
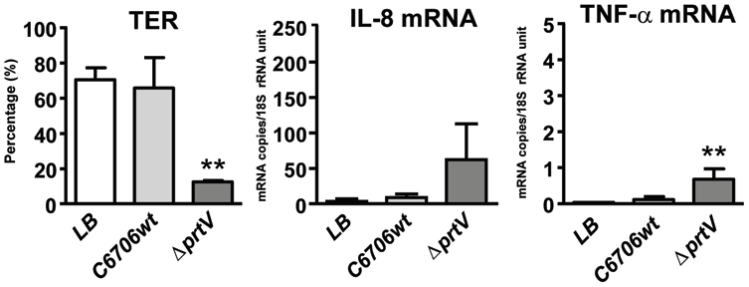
Tight monolayer responses to factors secreted by wt and *ΔprtV V. cholerae* in LB broth. Transepithelial electrical resistance (TER) (**a**) and IL-8 (**b**), and TNF-α (**c**) mRNA expression levels in polarized tight monolayers of T84 intestinal epithelial cells challenged with culture supernatants from wild type *V. cholerae* strain C6707 (C6706wt) and the PrtV deletion mutant (*ΔprtV*) cultured in LB broth at the apical side for 5 hours at 37°C. TER is expressed as percentage of TER before incubation with the supernatant, calculated as [(TER after 5 hours incubation/TER at onset of incubation) x 100] for the individual wells. Cytokine mRNA expression levels were determined by real-time quantitative RT-PCR with RNA copy standards and normalized to the amount of 18S rRNA in the respective sample, giving an estimate of the average number of mRNA copies per cell. Bars and whiskers represent mean +1SD of three independent experiments. Statistically significant changes from LB controls as estimated by two-sided Student's t-test are indicated. ** p-value <0.01.

Initially, the possibility that PrtV directly affects the intestinal epithelium was addressed. LB broth supernatants from overnight cultures of *V. cholerae* C6706 (C6706wt) and the PrtV deletion mutant (*ΔprtV*) [10] were added to the apical side of tight monolayers of polarized T84 cells as *in vitro* model for human intestinal epithelium. Permeability was assessed as transepithelial electrical resistance (TER) and the average expression levels/cell of mRNA for the pro-inflammatory cytokines IL-8, IL-6, and tumor necrosis factor-α (TNF-α) and the down-regulatory, wound-healing, and “epithelium-sealing” cytokine transforming growth factor-β1 (TGF-β1) were determined. TER stayed high in tight monolayers to which LB broth alone or culture supernatant from C6706wt had been added (Fig. 1a). In contrast, culture supernatants from *ΔprtV* caused a significant drop in TER, i.e., 12.5±1.1% of that in the LB broth control, suggesting a significant increase in permeability (Fig. 1a). mRNA expression levels in LB broth controls were low for IL-6 and TNF-α (mean±SD, 0.001±0.0001 and 0.037±0.006 mRNA copies/18S rRNA unit for IL-6 and TNF-α, respectively, n = 3), intermediate for IL-8 (4.6±4.3 mRNA copies/18S rRNA unit, n = 3) and high for TGF-β1 (33.6±5.2 mRNA copies/18S rRNA unit, n = 3). Supernatant from *ΔprtV* caused a more than 20-fold increase of IL-8 and TNF-α mRNA levels while supernatant from C6706wt caused only marginal increases both cytokines (Fig. 1b, c). In contrast, IL-6 and TGF-β1 mRNA levels did not change upon exposure to culture supernatants from *ΔprtV* or C6706wt (data not shown). Deletion of CTX or both CTX and PrtV did not cause any changes compared to C6706wt in capability to secrete factors that influenced permeability and/or cytokine mRNA levels in tight monolayers (data not shown).

### PrtV Does Not Inactivate Pro-Inflammatory Factor(s) when the Bacteria Are Cultured in Brain/Heart Infusion (BHI) Broth

**Figure 2:**
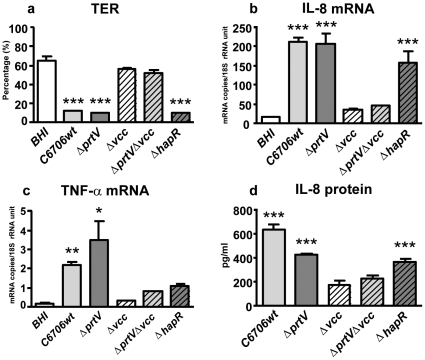
Tight monolayer responses to factors secreted by *V. cholerae* and mutant derivates in BHI broth. TER (**a**), expression levels of IL-8 (**b**) and TNF-α (**c**) mRNAs and amounts of IL-8 protein secreted (**d**) by polarized tight monolayer cells challenged with BHI broth culture supernatants from C6706wt, and deletion mutants for PrtV (*ΔprtV*), VCC (*Δvcc*), both PrtV and VCC (*ΔprtVΔvcc*) and HapR (*ΔhapR*). Challenge with supernatants and TER and cytokine mRNA level determinations as in Figure 1. IL-8 protein is given as the concentration determined by ELISA in the culture medium from the lower chamber of tight monolayers after subtraction of the concentration in the BHI control in the respective experiment. Bars and whiskers represent mean + 1SD of 3 independent experiments. Statistically significant changes from BHI controls as estimated by two-sided Student's t-test are indicated. * p-value ≤0.05, ** p-value <0.01, and *** p-value <0.001.

Unexpectedly, the supernatants from C6706wt caused a significant drop in TER (Fig. 2a) and increases in IL-8 and TNF-α mRNA levels (Fig. 2b, c) when the bacteria were cultured in BHI broth instead of LB (Fig. 2a–c). These changes were comparable to those of tight monolayers incubated with supernatants from *ΔprtV* cultured in BHI (Fig. 2a–c) or LB (Fig. 1a–c). The elevated IL-8 mRNA levels were accompanied by secretion of IL-8 protein to the culture medium at the basolateral side of the tight monolayers (Fig. 2d). The magnitude of these responses was similar to that of tight monolayer cells exposed to supernatants from *ΔprtV* cultured in LB, suggesting that BHI broth in some way inhibits PrtV degradation of the factor(s) causing the inflammatory response. Secreted TNF-α protein could not be detected by the same method (data not shown). In addition, culture supernatants of the non-01/non-0139 strain V11 caused a stronger inflammatory response when the bacterial strain was grown in BHI broth compared to LB broth (see Supplementary Text S1 and Figure S1) suggesting that this could be a universal feature of VCC secreting *Vibrio* strains. The stronger inflammatory response correlated with larger amounts of secreted VCC when the bacteria were grown in BHI broth (Figure S2).

### Experiments with Deletion Mutants Suggest that VCC Is the Factor Causing an Inflammatory Response in Epithelial Cells and that PrtV Is the Major Factor Causing Its Inactivation

Tight monolayers challenged with BHI broth supernatants from the VCC deletion mutant (*Δvcc*; Table 1) and the PrtV/VCC double deletion mutant (*ΔprtV/Δvcc*; Table 1) retained the same high TER as the BHI broth control while tight monolayers incubated with supernatants from *ΔprtV* showed a highly significant decrease in TER (Fig. 2a). Both IL-8 production (Fig. 2b,d) and TNF-α mRNA levels remained close to the levels of the BHI-broth control (Fig. 2b–d). These results suggest that VCC, but not PrtV, is the factor inducing an inflammatory response. That PrtV is the major component causing degradation of VCC is indicated by the fact that supernatants from a derivative carrying an in-frame deletion mutation in the gene coding for a regulator of *V. cholerae* protease expression (Δ*hapR*; Table 1) caused the same changes in TER and IL-8 production as *ΔprtV* (Fig. 2a,b,d), although several proteases in addition to PrtV are inactivated by deletion of the HapR gene. TGF-β1 and IL-6 mRNA levels were unchanged upon challenge with supernatants from wild type and mutant bacteria cultured in BHI (data not shown).

**Table 1:** Bacterial strains utilized.

Strain	Relevant genotype/phenotype	References
*V. cholerae* C6706	El Tor, Inaba, Strep^R^	28
V11	non-O1 non-O139 clinical isolate (2004)	Swedish Institute of Infectious Diseases
KVQ1	C6706 str2 Δ*hapR*	10
KVQ2	C6706 str2 Δ*luxO*	10
KAS122	C6706 str2 Δ*prtV*	10
BML30	C6706 str2 Δ*vcc*	This study
PKR1	C6706 str2 Δ*vcc* Δ*prtV*	This study
SNW50	C6706 *ctx::km*	This study
SNW51	C6706 *ctx:.km* Δ*prtV*	This study

### The PrtV Protease Affects *V. cholerae* Hemolytic Activity by Influencing VCC Stability

**Figure 3:**
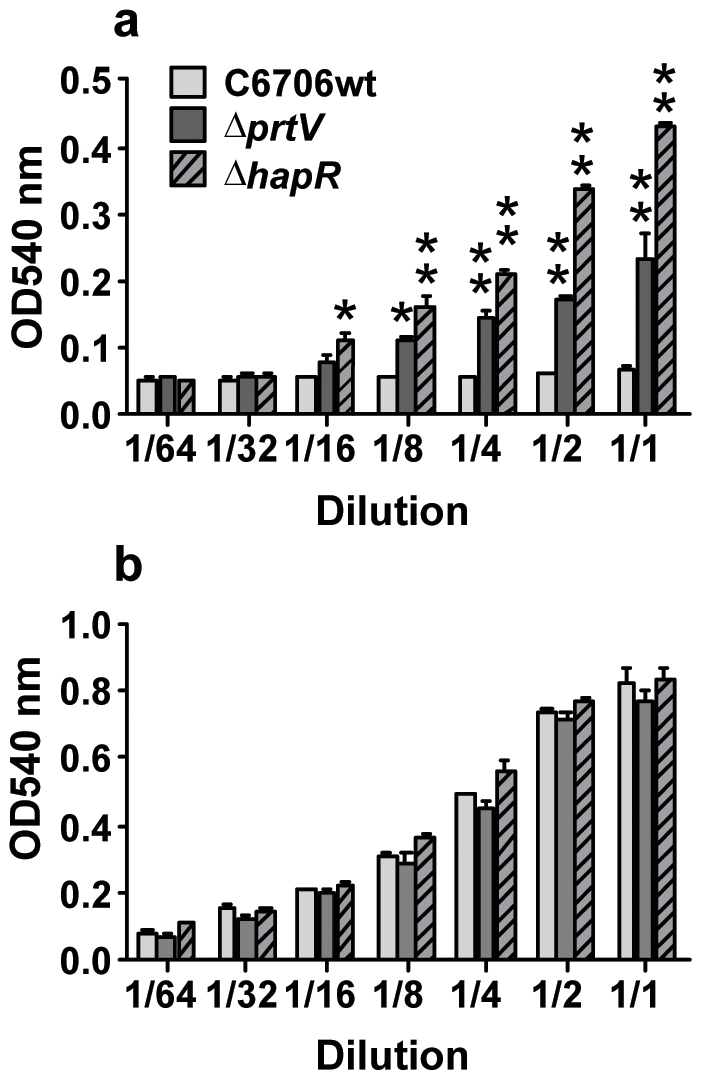
Hemolytic activity of secreted factors from wild type *V. cholerae* and deletion mutant derivates. Culture supernatants of C6706wt, *ΔprtV*, and *ΔhapR* were serially diluted and tested for hemolytic activity. (**a**) Bacteria grown in LB broth. (**b**) Bacteria grown in BHI broth. Bars and whiskers represent mean + 1SD of 3 independent experiments. Statistically significant changes from C6707wt at each dilution as estimated by one-way ANOVA are indicated. * p-value ≤0.05 and ** p-value <0.01.

Our results suggest that PrtV influences an inflammatory activity of VCC on epithelial cells. Therefore we tested whether PrtV would influence the hemolytic activity of VCC. Using supernatants of C6706wt, Δ*prtV* and Δ*hapR* grown in LB, we monitored the capacity to induce hemolysis of rabbit red blood cells. Hemolytic activity was enhanced in the case of mutant derivatives defective in the PrtV protease expression or the HapR regulator (Fig. 3a). These results suggested that PrtV protease modulated VCC hemolytic activity by means of proteolytic action. Culture supernatants from bacteria grown in BHI broth showed higher hemolytic activity in comparison to supernatants from bacteria grown in LB (Fig. 3b). In line with the epithelial cell tight monolayer experiments, the hemolytic activity of C6706wt was high, and no increase was seen with any of the mutants when the bacteria were grown in BHI broth, although a significant increase in hemolytic activity was observed when the bacteria were grown in LB (Fig. 3a,b). It remains to be analyzed how BHI broth can influence the synthesis of proteases in *V. cholerae*.

### *ΔprtV* Supernatant Contains More VCC than C6706wt Supernatant when Cultured in LB Broth

**Figure 4:**
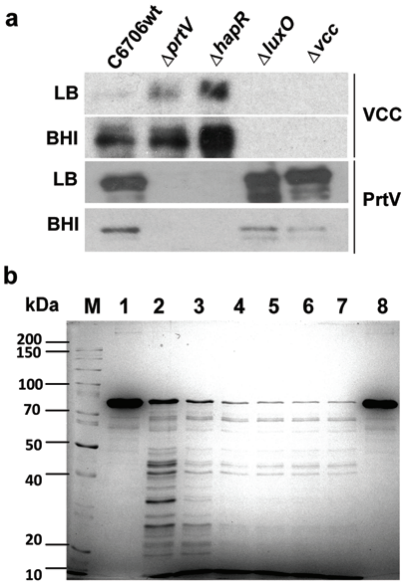
Analyses of VCC degradation by PrtV. (**a**) Immunoblot analyses of proteins from 1 ml samples of 24 hours culture supernatants of C6707wt, *ΔprtV*, *ΔhapR*, *ΔluxO* and *Δvcc* grown in BHI or LB broth as indicated using anti-VCC (upper rows) and anti-PrtV (lower rows) antisera. Three µg protein was applied to each lane. (**b**) SDS-PAGE and Coomassie blue staining of pure VCC protein (0.4 mg/ml) incubated with pure PrtV protease (50 nM) for 1 min (lane 2), 5 min (lane 3), 15 min (lane 4), 30 min (lane 5), 60 min (lane 6), and 120 min (lane 7). Lane 8: VCC incubated for 120 min without PrtV. (M)  =  protein molecular weight markers.

In order to analyze the secreted VCC levels of C6706wt and its derivatives, we performed immunoblot analyses using anti-VCC antiserum. The level of secreted PrtV was monitored using anti-PrtV antiserum. In the absence of the PrtV (*ΔprtV*) and the positive regulator of the secreted proteases, HapR (*ΔhapR*), the level of VCC was clearly increased compared with the wild type strain C6706 (C6706wt; Fig. 4a). Deletion of the gene encoding the LuxO regulator (*ΔluxO*) causes increased HapR levels in *V. cholerae*, resulting in overproduction of several proteolytic enzymes [10], [12] that could degrade the VCC cytolysin. Consistent with this, the VCC levels in *ΔluxO* were dramatically reduced both in LB and BHI media (Fig. 4a).

### VCC Alone Can Induce a Full-Fledged Inflammatory Response in Intestinal Epithelial Cells

**Figure 5:**
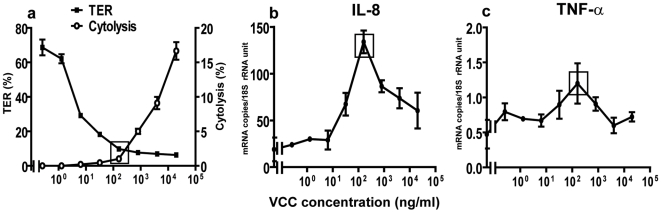
Effects of VCC on permeability, cell viability and cytokine levels in intestinal epithelium tight monolayers. TER (**a**, left y-axis), cytolysis (LDH release) (**a**, right y-axis), and IL-8 (**b**) and TNF-α (**c**) mRNA levels in polarized tight monolayer cells challenged with serial dilutions of pure VCC protein in LB broth at the apical side for 5 hours at 37°C. Results are from 3 independent experiments and presented as mean ±1 SD for each concentration. Rectangles indicate results at 160 ng VCC/ml.

The question of whether VCC could be a factor causing the inflammatory response in epithelial cells was addressed by incubating tight monolayers with serial dilutions of the purified VCC protein at concentrations from 0.26 ng/ml to 20 µg/ml. As little as 6.4 ng/ml VCC caused a significant drop in TER (Fig. 5a). VCC did not cause cell death, measured as lactate dehydrogenase (LDH) release, at this concentration, while significant cell death was seen at VCC concentrations of 800 ng/ml and above (Fig. 5a). The levels of IL-8 and TNF-α mRNAs increased with increasing amounts of VCC, up to 160 ng/ml, and reached the levels in tight monolayers incubated with supernatants of *ΔprtV* or C6706wt cultured in BHI (Fig. 5b,c). Increased IL-8 and TNF-α mRNA levels were inversely correlated to TER up to this point. At higher VCC concentrations, a decrease in cytokine mRNA levels was observed that paralleled the increase in cell death (Fig. 5a–c). VCC had no influence on IL-6 mRNA levels (not shown). Notably, at the VCC concentration where the IL-8 and TNF-α response was strongest, there was a marked drop in TER without any increase in cell death (Fig. 5a–c).

### VCC Is a Substrate for the PrtV Protease and PrtV Modulates the Pro-Inflammatory Effect of VCC

**Figure 6:**
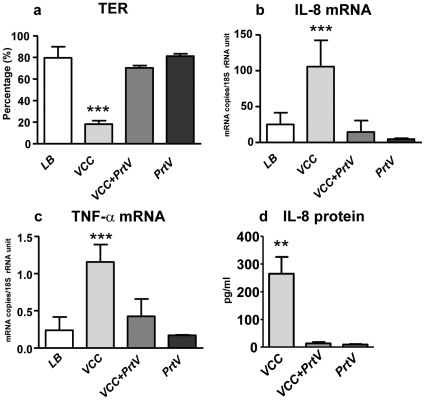
PrtV abolishes VCC induced increases in permeability and cytokine production in intestinal epithelium tight monolayers. TER (**a**) and IL-8 (**b**) and TNF-α (**c**) mRNA levels in polarized tight monolayer cells and amounts of IL-8 protein secreted (**d**) upon challenge with 160 ng/ml of pure VCC (VCC), VCC treated with PrtV, as in Figure 4b, for 30 min and thereafter diluted to 160 ng VCC/ml and 0.02 nM PrtV (VCC+PrtV), and 0.02 nM pure PrtV (PrtV) in LB broth. (LB)  =  LB broth control. Bars and whiskers represent mean + 1SD of 3 independent experiments. Statistically significant changes from LB broth controls are indicated. ** p-value <0.01, and *** p-value <0.001.

*In vitro* experiments with both PrtV and VCC included as purified proteins effectively ruled out the possibility that some other bacterial component could be responsible for cleavage of VCC. The VCC protein was rapidly digested into a series of polypeptides that appeared smaller than 45 kDa, and within 5 minutes the majority of the VCC protein was degraded (Fig. 4b). These results fully support the suggestion that PrtV is the major protease responsible for reducing the cytolytic and hemolytic activities of *V. cholerae*. Thus, PrtV may also moderate the inflammatory effects through degradation of VCC. This possibility was addressed by treating pure VCC protein with pure PrtV before addition to the polarized tight monolayers, using the VCC concentration that yielded optimal inflammatory effect. Monolayers incubated with VCC and PrtV only were run in parallel. TER, LDH release, IL-8 mRNA and TNF-α mRNA levels and amounts of secreted IL-8 protein were determined and compared to LB-broth controls. In line with previous experiments, VCC alone caused a significant drop in TER without measurable cell death (Fig. 6a and not shown), increased IL-8 mRNA and protein levels (Fig. 6b,d), and TNF-α mRNA levels (Fig. 6c). The effects on TER, IL-8, and TNF-α were all abolished by PrtV treatment (Fig. 6a–d). PrtV alone did not induce changes in any of these parameters (Fig. 6a–d). Thus, PrtV degradation of VCC seems to play a role in modulating reactogenicity of *V. cholerae*.

## Discussion

This study emerged from the previous, intriguing results of reverse molecular genetics experiments suggesting that the *Vibrio* protease PrtV confers an “anti-infammatory”, seemingly protective effect on human intestinal epithelium while it exerts a pathogenic effect in the intestine of the nematode *C. elegans* [10]. We found that PrtV executes its anti-inflammatory action by degrading secreted VCC and that BHI broth somehow protects VCC from degradation by PrtV. The latter finding was underscored by the consistent, parallel changes in capacity to induce an inflammatory response in intestinal epithelial cells and to cause lysis of red blood cells in bacterial culture supernatants depending of type of broth used, i.e. supernatants of wild type bacteria grown in LB caused little hemolysis and a low inflammatory response while supernatants of bacteria grown in BHI caused extensive hemolysis and a strong inflammatory response. The inflammatory response was characterized by increased epithelium permeability and production of IL-8 and low amounts of TNF-α by the epithelial cells. Experiments with pure VCC protein showed that VCC alone is likely to be responsible for these effects. Thus, VCC could be the causative agent of several symptoms induced by CTX-deficient strains of *V. cholerae*. In line with this notion, Debellis et al. [13] recently reported that VCC can cause decreased trans-tissue electrical resistance and increased chloride secretion when added at the luminal side of human colon mucosa specimens stripped of mucous layer.

VCC at concentrations above 160 ng/ml caused death of epithelial cells. However already at lower concentrations there were significant increases in permeability that could reflect paracellular leakage due to decreased tight junction stability. Since VCC acts by forming transmembrane pores, it is also possible that the epithelial cells lose cellular integrity due to difficulties in maintaining intracellular homeostasis. It is noteworthy that VCC transmembrane pores are too small (1.2 nm in inner diameter) to allow passage of proteins while ions easily could pass through [14].

VCC is an exotoxin produced by most O1 biotype El Tor and non-O1/non-O139 *V. cholerae* isolates [9], [15]. It is secreted as an inactive 80 kDa pro-toxin and processed by proteolytic cleavage into a 65 kDa active cytolysin [16]–[18], which can assemble into transmembrane pores in eukaryotic cells [14]. This toxin causes vacuolization, necrosis, or autophagy depending on the cell type and toxin concentration [19]–[24]. The VCC protein is rapidly lethal for mice after intravenous administration [8]. Olivier *et al.* described VCC as a causal factor of the tissue damage that occurs in the small intestinal epithelium in an adult mouse infection model [8]. Our results confirm that VCC efficiently lyses red blood cells and show that VCC also can kill human intestinal epithelial cells in polarized tight monolayers. Thus, VCC might cause serious damage both at the intestinal epithelial lining and in the blood. Notably, lower non-toxic doses of VCC induced an inflammatory response in intestinal epithelial cells that included increased epithelial permeability and induction of IL-8 and TNF-α, This suggests that intestinal epithelial cells initially execute a front-line defense to *V. cholerae* infection which is induced by VCC and involves recruitment of immune cells to the infected site by IL-8 and activation of epithelial cells, intraepithelial lymphocytes, and lamina propria macrophages by TNF-α. In this system, VCC seems to account for virtually all of the stimulation caused by *V. cholerae* secreted factors. It has been proposed that VCC has a role in the pathogenesis of gastroenteritis, particularly in strains that do not produce CTX [25]. Our results are compatible with this notion, although it is not known to what extent an epithelial inflammatory response contributes to the symptoms. CTX, which clearly is involved in cholera diarrhea, did not contribute to the inflammatory response in the tight monolayer cells, which is in agreement with a previous report [26]. It is important to keep in mind that the intestinal epithelial cells are polarized and receptors for bacterial components like the TLRs are preferentially localized to the basolateral side *in vivo* [27]. This is also likely to be the case in the *in vitro* model used here. Thus, once the bacterium and/or its released factors have penetrated the epithelial barrier into the tissues, additional mechanisms may come into play.

The present finding that the inflammatory response is almost completely abolished by PrtV degradation of VCC suggests that VCC is indeed an autologous substrate for PrtV and that it is involved in modulation of *V. cholerae* reactogenicity. The reason the bacterium modulates its toxicity at the level of secreted proteins rather than at the transcriptional level is not known. It might be that this is an adaptation to achieve the best strategy for different environmental conditions. In line with this notion, VCC degradation by PrtV was inhibited by yet undefined factor(s) in the more complex bacterial culture media. Thus, VCC was spared from proteolysis when wild type *V. cholerae* was grown in BHI broth but degraded when the bacteria were grown in LB. Accordingly, the capacity to induce an inflammatory response in tight monolayer cells and to lyse red blood cells was only observed when the bacteria were grown in BHI medium or were PrtV-deficient. BHI seems to influence the overall functional state of the bacteria since the total amount of secreted *V. cholerae* proteases is reduced in BHI broth (not shown). Furthermore, BHI could contain a chelator, depriving the medium of divalent ions, thereby inactivating PrtV, and/or be rich in other substrates for PrtV proteolysis, thereby sparing VCC. The content of the gut lumen is likely to be more similar to BHI than to LB and consequently degradation of VCC would be minimal, which would give the opportunity for VCC to exert its toxic effects at the epithelial lining and subsequently allow access of the bacteria to the bodily tissues.

## Materials and Methods

### Bacterial Strains

Bacterial strains and mutant constructions used are listed in Table 1. A Δ*vcc* mutant was constructed by crossover PCR essentially as described previously [10], [12], [28]. Briefly, a 500 bp 5′ flanking sequence of the gene including several nucleotides of the coding region was PCR amplified with the primers *vcc*-A (5′-CGCTCTAGACCTTGGAGTTGCAGGTAGGC-3′) and *vcc*-B (5′-TGTTAGTTTATAGTGGATGGGGGTTGGACTGGATATTGCGC-3′). The primers *vcc*-C (5′-CCCATCCACTATAAACTAACAGCAAGCTTGCAACGTCTCTC-3′) and *vcc*-D (5′-CGCTCTAGACCTTGTTGCGATAACGCGTGA-3′) were used to amplify several nucleotides of the 3′ region of the gene plus 500 bp of the downstream flanking sequence. The two PCR products were annealed at their overlapping region and amplified by PCR to produce a DNA single fragment, using the outer primers (*vcc*-A and *vcc*-D). The resulting PCR product, lacking most of the coding sequence of the gene, was digested with *Xba*I enzyme and ligated into similarly a digested pCVD442 suicide plasmid. The pCVD442::Δvcc was introduced into *E. coli* SM10λ*pir* by electroporation. The donor *E. coli* S10λ*pir* containing the plasmid pCVD442::Δ*vcc* was used for conjugal transfer to a rifampicin-resistant *V. cholerae* O1 El Tor C6706 strep^R^ or Δ*prtV* strain. A mixture containing equal volumes of the donor and recipient in LB was incubated for 6 h at 30°C. The exconjugants were selected by plating the suspension onto LB plates supplemented with 100 µg/ml of streptomycin (strep) and 100 µg/ml of carbenicillin (Cb) and incubated at 30°C. After selection of the desired transconjugants on strep+Cb plates, they were streaked onto LB-plates with 10% sucrose and incubated at 30°C. Several colonies were purified from the plates, tested for Cb sensitivity and then analyzed for the deletion by using colony PCR.

The *ctx*::km and *ctx*::kmΔ*prtV* mutant were obtained by phage-mediated transduction using the CP-T1ts phage [29]. The CP-T1ts stock was obtained from Dr. Andrew Camilli. Bacteriophage CP-T1ts propagated in a strain derived from E7946::km were prepared by the plate method. *V. cholerae* cells were grown to late-exponential phase (OD 600∼0.7), and infected at a multiplicity of infection of 10^−5^ with CP-T1ts. Bacteriophage were allowed to adsorb 10 min at room temperature, then mixed with 5 ml of 45°C LB soft agar (0.5%) and poured onto LB agar plates. The plates were incubated at 30°C. Bacteriophage were recovered from the soft agar layer of plates by resuspending the top agar in 5 ml of LB broth and incubated at 4°C to allow bacteriophage diffusion. Agar and bacteria were removed by two successive centrifugations at 15,000 x g for 1 min. Bacteriophage were concentrated by centrifugation at 16,000 x g for 2 h at 4C. The pellet containing bacteriophage were suspended in 500 µl of LB broth and used for transduction. Phage transduction was done as follows: the recipient strains (C6706 and Δ*prtV*) were grown in 10 ml LB to late-exponential phase (OD 600∼0.7), spun down and resuspended in 1 ml LB broth. One half ml of the bacterial suspension, 0.5 ml of phage suspension and 0.5 ml of adsorption buffer (0.015 M CaCl_2_ + 0.03 M MgCl_2_) were mixed and incubated at 30°C for 20 min. The cells were washed once in 0.9% NaCl, spun down and resuspended in 0.3 ml of NaCl. One hundred µl samples of the suspension were spread out on plates containing 30 µg/ml kanamycin (Sigma). Transductant colonies were purified and used for further experiments.

Bacteria were grown overnight at 37°C with shaking in 20 ml of LB or BHI broth supplemented, as appropriate, with kanamycin (30 µg/ml) or ampicillin (50 µg/ml).

### Bacterial Proteins

The purification of VCC and PrtV from *V. cholerae* was described in [18] and [11], respectively. To test the proteolytic activity of PrtV on VCC, pure VCC protein (0.4 mg/ml) was incubated with 100 nM pure PrtV protein in 50 mM HEPES, 1 mM CaCl_2_ and 1 mM MgCl_2_ (pH 7.2) at 37°C.

### Intestinal Epithelial Cell Tight Monolayer Cultures

Polarized tight monolayers of the established human colon carcinoma cell line T84 (American Type Culture Collection, Rockville, MD, USA) were established on semi-permeable membrane supports as described [30]. Tissue culture medium in the upper chamber was replaced by culture supernatants from different bacterial strains grown in LB or BHI broth or serial dilutions of VCC and PrtV proteins in LB when a TER of ≥1,000 Ohm/cm^2^ was obtained. Monolayers were thereafter incubated at 37°C in 5% CO_2_ for 5 hours, at which time TER was measured, culture supernatants were collected for LDH release and cytokine protein analysis, and tight monolayer cells were collected in 4 M guanidinium thiocyanate with 25 mM sodium citrate, 0.5% N-laurosylsarcosine and 0.1 M 2-mercaptoethanol (pH 7.0) for RNA extraction.

### Determination of Cytokine mRNA Expression Levels

Total RNA was extracted as described [30] and stored at −80°C. Concentrations of IL-6, IL-8, TNF-α and TGF-β1 mRNAs were determined in triplicate by real-time quantitative RT-PCR assays with RNA copy standards as described [30]–[32]. The 18S rRNA content was determined in all samples using a real-time quantitative RT-PCR kit (Applied Biosystems) and results were expressed as mRNA copies per 18S rRNA unit.

### Determination of Secreted Cytokines

Concentrations of IL-8 and TNF-α proteins were determined in duplicates of tissue culture medium collected from the lower chamber of monolayer cultures by using the Endogen Human IL-8 and TNF-α ELISA Kits, respectively (Pierce Biotechnology, Rockford, IL). Results are given as the concentration determined by ELISA in culture medium from the lower chamber of tight monolayer cultures after subtraction of the concentration in the broth control in the respective experiment.

### Cytolysis Assay

Cell death in monolayer cultures was estimated as the LDH activity released to the lower chambers using the Cytotoxicity Detection Kit (LDH) (Roche Applied Science, Mannheim, Germany). Cytolysis (%) was calculated as: [(experimental OD_405_ value - low control OD_405_ value)/(high control OD_405_ value - low control OD_405_ value)] ×100. “Low control” is the spontaneous release of LDH from parallel tight monolayers in complete culture medium and “high control” the LDH activity in supernatants from parallel tight monolayers incubated in the presence of 2% Triton X100.

### Hemolytic Activity Assay

Hemolysis was estimated as release of hemoglobin as described [34]. Rabbit red blood cells (8%) were suspended in PBS containing 0.1% gelatin. Fifty µl of the suspension was loaded into the wells of a 96-well microtiter plate containing 50 µl of serially diluted bacterial culture supernatants. The microtiter plate was incubated at 37°C for 120 min and subsequently centrifuged at 1800 x *g* for 10 min. Fifty µl from each well was transferred to a new plate and the absorbance at 540 nm was measured spectrophotometrically.

### SDS-PAGE and Western Blot Analysis

Bacterial strains were grown in LB and BHI media with aeration by shaking at 30°C for 24 h. Bacteria were harvested by centrifugation at 10,000 x g for 10 min at 4°C. The culture supernatant fluid was precipitated with 10% trichloroacetic acid (TCA). Briefly, 1 volume (250 µl) of 50% TCA was added to 4 volumes (1 ml) of protein sample. The protein-TCA mixture was kept on ice for 15 min, and subsequently, the tube was centrifuged at 15,000 x g for 5 min. The supernatant was removed, the protein pellet was washed with 200 µl of cold acetone, and the tube was centrifuged at 15,000 x g for 5 min. The resulting pellets were dissolved in SDS sample buffer and the protein samples were subjected to polyacrylamide gel electrophoresis and then blotted onto a PVDF membrane [34]. Proteins were identified using anti-VCC [19] and anti-PrtV [10] polyclonal rabbit antisera at a final dilution of 1∶10,000 and 1∶200,000 respectively. Anti-rabbit Ig horseradish peroxidase-conjugate (GE Healthcare) was used as a secondary antibody at a final dilution of 1∶20,000. The ECL+ chemiluminescence system (GE Healthcare) was used to detect the level of chemiluminescence that was then monitored using a Fluor-S MultiImager (BioRad) and by autoradiography.

### Statistical Analyses

Statistical analysis of changes in TER, LDH release, cytokine mRNA expression levels and IL-8 protein concentrations caused by bacterial culture supernatants and the VCC and PrtV proteins compared to LB or BHI broth controls was performed using Student's t-test. Statistical significance of changes in hemolytic activity of *ΔprtV* and *ΔhapR* compared to C6706wt was evaluated using one-way ANOVA with Dunnett's post-test at each dilution. Two-sided assays were used throughout. Differences with a p-value ≤0.05 were considered statistically significant. Results are given as mean±1SD.

## Supporting Information

Figure S1 Effects of *V. cholerae* V11 strain culture supernatants on permeability and IL-8 mRNA expression. Supernatants from 24 hours cultures of non-01/non-0139 *V. cholerae* strain V11 grown in LB broth (LB) or BHI broth (BHI) were added to the apical side of polarized tight monolayers of T84 cells. Changes in transepithelial electrical resistance (TER) as percent of TER at onset of incubation (a), cell death determined as percent of released LDH (b), expression levels of IL-8 (c) and TNF-α (d) mRNAs as determined by real-time quantitative RT-PCR were monitored after 5 hours incubation. Bars and whiskers indicate mean + 1SD of three independent experiments. Black insert bars in (c) and (d) indicates the cytokine mRNA levels in LB and BHI broth controls, respectively.
(0.41 MB TIF)

Figure S2 Immunoblot analyses of VCC and PrtV of *V. cholerae* grown in LB and BHI media. *V. cholerae* O1 strain C6706 (C6706), non-O1/non-O139 strain V11 (V11) and C6706 strain VCC deletion mutant (Δvcc) were grown in LB and BHI media for 24 hours and proteins from 1 ml culture supernatants were analysed by immunoblot using anti-VCC and anti-PrtV polyclonal antisera.
(0.29 MB TIF)

Text S1 Supplementary Text S1
(0.03 MB DOC)
