# Supplementary figures and images for: Vibrio cholerae Cytolysin Causes an Inflammatory Response in Human Intestinal Epithelial Cells That Is Modulated by the PrtV Protease
Source: PLoS One. 2009 Nov 12;4(11):e7806. doi: 10.1371/journal.pone.0007806 (PMC2771358; doi:10.1371/journal.pone.0007806)

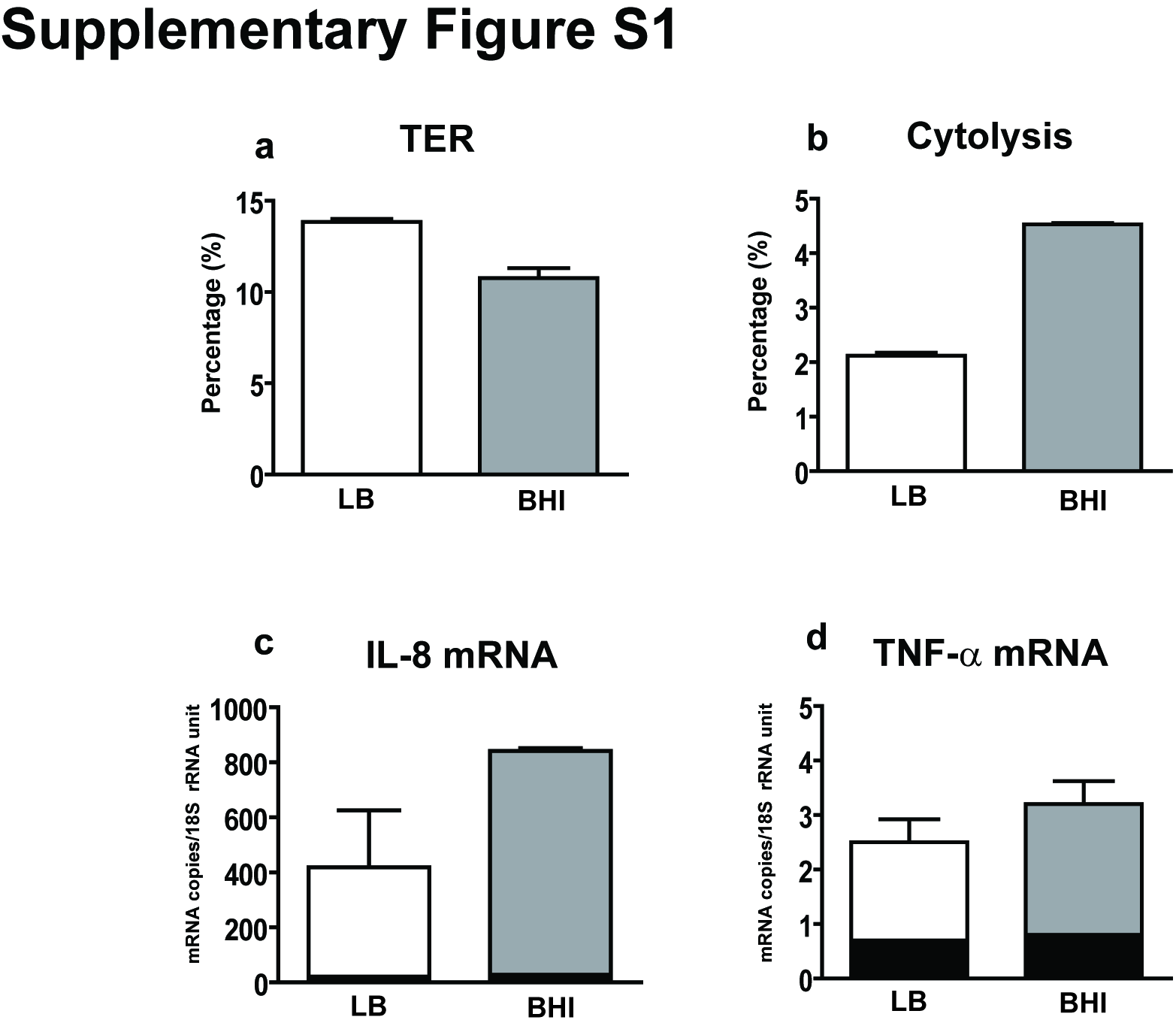

Supplement: Figure S1 — Effects of V. cholerae V11 strain culture supernatants on permeability and IL-8 mRNA expression. Supernatants from 24 hours cultures of non-01/non-0139 V. cholerae strain V11 grown in LB broth (LB) or BHI broth (BHI) were added to the apical side of polarized tight monolayers of T84 cells. Changes in transepithelial electrical resistance (TER) as percent of TER at onset of incubation (a), cell death determined as percent of released LDH (b), expression levels of IL-8 (c) and TNF-α (d) mRNAs as determined by real-time quantitative RT-PCR were monitored after 5 hours incubation. Bars and whiskers indicate mean + 1SD of three independent experiments. Black insert bars in (c) and (d) indicates the cytokine mRNA levels in LB and BHI broth controls, respectively. (0.41 MB TIF) [file pone.0007806.s001.tif]

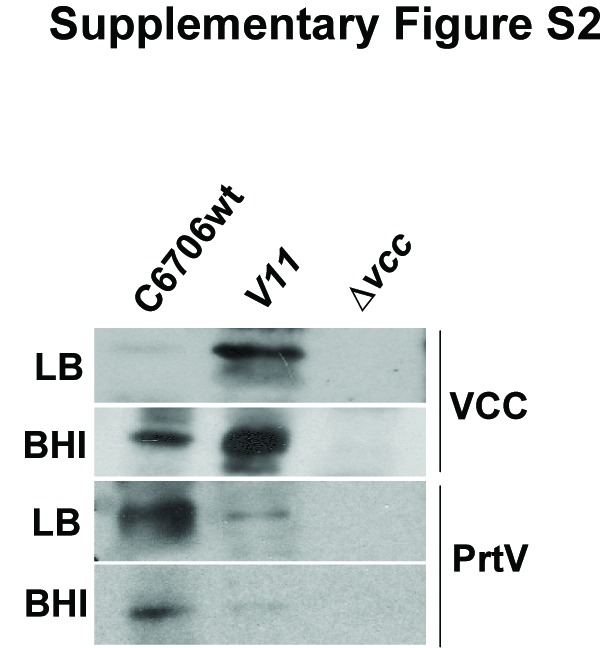

Supplement: Figure S2 — Immunoblot analyses of VCC and PrtV of V. cholerae grown in LB and BHI media. V. cholerae O1 strain C6706 (C6706), non-O1/non-O139 strain V11 (V11) and C6706 strain VCC deletion mutant (Δvcc) were grown in LB and BHI media for 24 hours and proteins from 1 ml culture supernatants were analysed by immunoblot using anti-VCC and anti-PrtV polyclonal antisera. (0.29 MB TIF) [file pone.0007806.s002.tif]
