# Supplementary material for: Vibrio cholerae Cytolysin Causes an Inflammatory Response in Human Intestinal Epithelial Cells That Is Modulated by the PrtV Protease
Source: PLoS One. 2009 Nov 12;4(11):e7806. doi: 10.1371/journal.pone.0007806 (PMC2771358; doi:10.1371/journal.pone.0007806)
Supplement: Text S1 — Supplementary Text S1 (0.03 MB DOC) [file pone.0007806.s003.doc]

**A stronger inflammatory effect on intestinal epithelial tight monolayer cells by culture supernatants of bacteria grown in BHI broth compared to LB broth seems to be a general phenomenon for *Vibrio* strains**

Twenty-four hours culture supernatants of the *V. cholerae* non-01/non-0139 strain V11 grown in LB and BHI broth were analysed for capacity to induce changes in permeability (TER), cell viability (release of LDH), and expression levels of mRNA for IL-8 and TNF-α in tight monolayer cells. As was the case for the C6706 wild-type strain, supernatant from V11 grown in BHI caused markedly higher increases in permeability and IL-8 mRNA expression levels than LB supernatants (Figure S1 a, c). However, the overall responses to supernatants of the V11 strain were higher than to supernatants of the C6706 strain grown in both broths (compare Figure S1 and Figures 1 and 2) suggesting that higher amounts of VCC are released from the V11 strain. It was confirmed by the findings that both BHI and LB supernatants of the V11 strain caused significant cell death (Figure S1 b) while supernatants of C6706wt did not and that the amounts of VCC is higher and the amounts of PrtV is lower in supernatants of the V11 strain compared to the C6706 strain in both LB and BHI broth as demonstrated by immunoblots (Figure S2).
